# Exploring the Impact of Wildfires on Children’s Psychological Well-being: A Comprehensive Review of Recent Literature

**DOI:** 10.1192/j.eurpsy.2024.953

**Published:** 2024-08-27

**Authors:** M. K. Adu, B. Agyapong, V. I. O. Agyapong

**Affiliations:** ^1^Department of Psychiatry, Dalhousie University, Halifax NS; ^2^Department of Psychiatry, University of Alberta, Edmonton, AB, Canada

## Abstract

**Introduction:**

Wildfire disasters have become increasingly rampant. There is a critical need for all to fully understand the mechanism and impact of these disasters on humans, with a special emphasis on the mental health effects they pose on the affected individuals and communities. This article specifically presents a scoping review of the psychological reactions of children and adolescents post-wildfire disaster.

**Objectives:**

This review aims to synthesize currently available literature regarding the impact of wildfire on mental health, specifically the psychological reactions of children to wildfires.

**Methods:**

We identified 8 research articles using 6 databases for this review. Data extraction was performed using a qualitative descriptive approach.

**Results:**

The results identified post-traumatic stress disorder (PTSD), anxiety, depression, stress, alcohol/substance misuse, hopelessness, low resilience, reduced quality of life, and self-esteem as the psychological conditions manifesting in children and adolescents post-wildfire disaster. PTSD was the most evaluated psychological reaction in the participants (7 out of eight studies).

**Conclusions:**

This review highlights that deleterious mental health effects, such as PTSD, depression, anxiety, and suicidality, can persist in children for years post-wildfire disaster. Factors such as gender, direct exposure to the wildfire, re-traumatization, and resilience informed or ameliorated the severity of the impact of wildfire on children and adolescents. Our findings further emphasize the need for multi-year funding and programs to support children and adolescents’ mental health, including children with disabilities in the communities that have experienced wildfire disasters.

**Disclosure of Interest:**

None Declared

